# Bis{*N*-[5-(4-methoxy­phen­yl)-1,3,4-oxa­diazol-2-yl]ethanimidamidato}copper(II)

**DOI:** 10.1107/S1600536810009050

**Published:** 2010-03-17

**Authors:** Yacine Djebli, Salima Mosbah, Sihem Boufas, Leila Bencharif, Thierry Roisnel

**Affiliations:** a Laboratoire de Chimie des Materiaux, Université Mentouri, 25000 Constantine, Algeria; b Sciences Chimiques de Rennes (UMR CNRS 6226), Université de Rennes 1, Avenue du Général Leclerc, 35042 Rennes Cedex, France

## Abstract

The title compound, [Cu(C_11_H_11_N_4_O_2_)_2_], was prepared by solvothermal synthesis using 2-amino-5-(4-methoxy­phen­yl)-1,3,4-oxadiazole and copper sulfate penta­hydrate in an acetonitrile solution. The Cu^II^ atom lies on an inversion center and is four-coordinated in a slightly distorted square-planar geometry by four N atoms of the ligands obtained from the formation of a bond between the amine N atom of the oxadiazole mol­ecule and the nitrile C atom of the solvent. In the crystal structure an inter­molecular N—H⋯N hydrogen bond links inversion-related mol­ecules.

## Related literature

For comparative bond lengths in similar coordination compounds, see: Cai, (2009 ▶). For applications of complexes formed by Schiff base ligands, see: Lu & Schauss (2002 ▶). For chemotherapeutic effects of 2,5-substituted-1,3,4-oxadiazole derivatives, see: Cao *et al.* (2002 ▶); Kadi *et al.* (2007 ▶); Zareef *et al.* (2006 ▶, 2007 ▶, 2008 ▶).
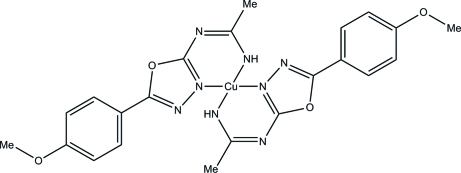


## Experimental

### 

#### Crystal data

[Cu(C_11_H_11_N_4_O_2_)_2_]
*M* *_r_* = 526.02
Triclinic, 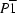

*a* = 4.9020 (6) Å
*b* = 11.2083 (14) Å
*c* = 11.5739 (14) Å
α = 111.501 (5)°
β = 99.274 (6)°
γ = 91.564 (5)°
*V* = 581.33 (12) Å^3^
*Z* = 1
Mo *K*α radiation
μ = 0.99 mm^−1^
*T* = 120 K
0.53 × 0.23 × 0.07 mm

#### Data collection

Bruker APEX diffractometer
Absorption correction: multi-scan (*SADABS*; Sheldrick, 2002 ▶) *T*_min_ = 0.707, *T*_max_ = 0.933
6654 measured reflections
2633 independent reflections
2417 reflections with *I* > 2σ(*I*)
*R*_int_ = 0.035

#### Refinement

*R*[*F* ^2^ > 2σ(*F* ^2^)] = 0.037
*wR*(*F* ^2^) = 0.087
*S* = 1.06
2633 reflections
166 parameters
H-atom parameters constrained
Δρ_max_ = 0.42 e Å^−3^
Δρ_min_ = −0.29 e Å^−3^

### 

Data collection: *SMART* (Bruker, 2002 ▶); cell refinement: *SAINT* (Bruker, 2002 ▶); data reduction: *SAINT*; program(s) used to solve structure: *SIR97* (Altomare *et al.*, 1999 ▶); program(s) used to refine structure: *SHELXL97* (Sheldrick, 2008 ▶); molecular graphics: *ORTEP-3 for Windows* (Farrugia, 1997 ▶); software used to prepare material for publication: *WinGX* (Farrugia, 1999 ▶).

## Supplementary Material

Crystal structure: contains datablocks I, global. DOI: 10.1107/S1600536810009050/pk2228sup1.cif

Structure factors: contains datablocks I. DOI: 10.1107/S1600536810009050/pk2228Isup2.hkl

Additional supplementary materials:  crystallographic information; 3D view; checkCIF report

## Figures and Tables

**Table 1:** Hydrogen-bond geometry (Å, °)

*D*—H⋯*A*	*D*—H	H⋯*A*	*D*⋯*A*	*D*—H⋯*A*
N1—H1⋯N8^i^	0.88	2.42	2.983 (2)	123

Symmetry code: (i) 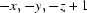.

**Fig. 1.**
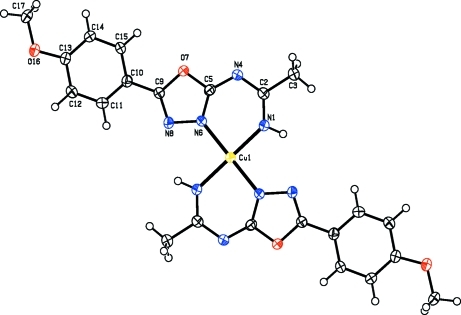
The molecular structure of the title compound in 30% probability displacement ellipsoids for non-H atoms.

## supplementary crystallographic information

### Comment

In recent years, there has been a considerable effort towards preparation of new materials containing polyfunctional organic ligands able to bind metallic ions by solvothermal synthesis. For example, with Schiff bases ligands, such complexes could be applied in different areas, including biochemistry, electrochemistry, and catalysis (Lu *et al.*, 2002).

The 2,5-substituted-1,3,4-oxadiazole derivatives are of significant interest due to their chemotherapeutic effects (Kadi *et al.*, 2007; Zareef *et al.*, 2008; Zareef *et al.*, 2007; Zareef *et al.*, 2006; Cao *et al.*, 2002). In this paper, we report the structure of one of these compounds with copper (II).

In the centrosymmetric title complex, the Cu (II) atom is located on an inversion center and is four-coordinated in a square planar geometry by four N atoms of the ligands obtained from the formation of a bond between *N*-amine of the oxadiazole molecule and *C*-nitrile of the solvent. All the coordinated bond lengths are typical and comparable with those in similar copper (II) complexes (Cai,2009). In the title compound, there is just one weak hydrogen bond N1-H1···N8 linking different inversion (-x, -y, -Z+1) related molecules.

### Experimental

5-(4-Methoxy-phenyl)-2amino-1, 3, 4-oxadiazole (0,2 mmole) and (0,1 mmole) copper sulfate pentahydrate were mixed in 5 ml of acetonitrile. The mixture was placed in a Teflon-lined stainless steel vessel, and heated to 160° C for 16 h. It was then cooled to room temperature over a period of 24 h, and washed using acetonitrile.  Brown crystals suitable for X-Ray crystallography were obtained.

### Refinement

H atoms were placed at calculated positions (C-H = 0..88-0.98 Å) and were treated as riding on their parent atoms, with U_iso_(H) set to 1.2-1.5 times U_eq_(C).

### Crystal data

**Table:**

[Cu(C_11_H_11_N_4_O_2_)_2_]	*Z* = 1
*M**_r_* = 526.02	*F*(000) = 271
Triclinic, *P*1	*D*_x_ = 1.503 Mg m^−^^3^
Hall symbol: -P 1	Mo *K*α radiation, λ = 0.71073 Å
*a* = 4.9020 (6) Å	Cell parameters from 2925 reflections
*b* = 11.2083 (14) Å	θ = 3.2–27.4°
*c* = 11.5739 (14) Å	µ = 0.99 mm^−^^1^
α = 111.501 (5)°	*T* = 120 K
β = 99.274 (6)°	Plate, brown
γ = 91.564 (5)°	0.53 × 0.23 × 0.07 mm
*V* = 581.33 (12) Å^3^	

### Data collection

**Table:**

Bruker APEXII diffractometer	2633 independent reflections
Radiation source: Enraf-Nonius FR590	2417 reflections with *I* > 2σ(*I*)
graphite	*R*_int_ = 0.035
CCD rotation images, thin slices scans	θ_max_ = 27.5°, θ_min_ = 3.2°
Absorption correction: multi-scan (*SADABS*; Sheldrick, 2002)	*h* = −6→6
*T*_min_ = 0.707, *T*_max_ = 0.933	*k* = −14→14
6654 measured reflections	*l* = −15→15

### Refinement

**Table:**

Refinement on *F*^2^	Primary atom site location: structure-invariant direct methods
Least-squares matrix: full	Secondary atom site location: difference Fourier map
*R*[*F*^2^ > 2σ(*F*^2^)] = 0.037	Hydrogen site location: inferred from neighbouring sites
*wR*(*F*^2^) = 0.087	H-atom parameters constrained
*S* = 1.06	*w* = 1/[σ^2^(*F*_o_^2^) + (0.0355*P*)^2^ + 0.2373*P*] where *P* = (*F*_o_^2^ + 2*F*_c_^2^)/3
2633 reflections	(Δ/σ)_max_ < 0.001
166 parameters	Δρ_max_ = 0.42 e Å^−^^3^
0 restraints	Δρ_min_ = −0.29 e Å^−^^3^

### Fractional atomic coordinates and isotropic or equivalent isotropic displacement parameters (Å^2^)

**Table:**

	*x*	*y*	*z*	*U*_iso_*/*U*_eq_	
Cu1	0	0	0.5	0.02231 (12)	
N1	0.1428 (3)	−0.09664 (16)	0.34848 (16)	0.0245 (4)	
H1	0.0696	−0.1769	0.3098	0.029*	
C2	0.3294 (4)	−0.06281 (19)	0.29405 (19)	0.0240 (4)	
C3	0.3968 (5)	−0.1574 (2)	0.1722 (2)	0.0314 (5)	
H3A	0.3138	−0.2438	0.1562	0.047*	
H3B	0.5986	−0.1581	0.1794	0.047*	
H3C	0.3216	−0.1314	0.1021	0.047*	
N4	0.4749 (3)	0.05335 (16)	0.33696 (16)	0.0255 (4)	
C5	0.4203 (4)	0.14395 (19)	0.44061 (19)	0.0229 (4)	
N6	0.2379 (3)	0.14792 (15)	0.51450 (16)	0.0230 (4)	
O7	0.5775 (3)	0.26005 (13)	0.48592 (13)	0.0244 (3)	
N8	0.2771 (3)	0.26958 (16)	0.61315 (17)	0.0257 (4)	
C9	0.4788 (4)	0.33114 (19)	0.59302 (19)	0.0234 (4)	
C10	0.6076 (4)	0.46056 (19)	0.6693 (2)	0.0251 (4)	
C11	0.5447 (5)	0.5265 (2)	0.7888 (2)	0.0368 (5)	
H11	0.417	0.4865	0.8202	0.044*	
C12	0.6665 (5)	0.6494 (2)	0.8615 (2)	0.0385 (6)	
H12	0.6239	0.6931	0.9429	0.046*	
C13	0.8519 (4)	0.70962 (19)	0.8158 (2)	0.0266 (4)	
C14	0.9179 (5)	0.6449 (2)	0.6975 (2)	0.0309 (5)	
H14	1.0455	0.6849	0.6661	0.037*	
C15	0.7950 (5)	0.5211 (2)	0.6259 (2)	0.0321 (5)	
H15	0.8404	0.4767	0.5452	0.038*	
O16	0.9576 (3)	0.83100 (14)	0.89471 (14)	0.0336 (4)	
C17	1.1494 (5)	0.8972 (2)	0.8518 (2)	0.0321 (5)	
H17A	1.3138	0.8496	0.8384	0.048*	
H17B	1.205	0.9841	0.9154	0.048*	
H17C	1.0601	0.903	0.7722	0.048*	

### Atomic displacement parameters (Å^2^)

**Table:**

	*U*^11^	*U*^22^	*U*^33^	*U*^12^	*U*^13^	*U*^23^
Cu1	0.02216 (19)	0.01886 (19)	0.0244 (2)	−0.00107 (13)	0.00942 (14)	0.00450 (14)
N1	0.0251 (8)	0.0203 (8)	0.0251 (9)	−0.0016 (6)	0.0080 (7)	0.0039 (7)
C2	0.0251 (10)	0.0244 (10)	0.0225 (10)	0.0037 (8)	0.0058 (8)	0.0083 (8)
C3	0.0365 (12)	0.0275 (11)	0.0263 (11)	−0.0012 (9)	0.0110 (9)	0.0039 (9)
N4	0.0290 (9)	0.0231 (9)	0.0238 (9)	−0.0008 (7)	0.0106 (7)	0.0057 (7)
C5	0.0225 (9)	0.0206 (10)	0.0261 (10)	−0.0003 (7)	0.0048 (8)	0.0094 (8)
N6	0.0233 (8)	0.0199 (8)	0.0238 (8)	0.0002 (6)	0.0094 (7)	0.0040 (7)
O7	0.0277 (7)	0.0197 (7)	0.0247 (7)	−0.0024 (5)	0.0093 (6)	0.0056 (6)
N8	0.0266 (9)	0.0187 (8)	0.0279 (9)	−0.0001 (7)	0.0097 (7)	0.0027 (7)
C9	0.0247 (10)	0.0208 (10)	0.0243 (10)	0.0022 (8)	0.0081 (8)	0.0064 (8)
C10	0.0257 (10)	0.0203 (10)	0.0274 (11)	0.0015 (8)	0.0068 (8)	0.0060 (8)
C11	0.0446 (13)	0.0299 (12)	0.0344 (12)	−0.0070 (10)	0.0200 (11)	0.0061 (10)
C12	0.0540 (15)	0.0298 (12)	0.0281 (12)	−0.0058 (10)	0.0201 (11)	0.0021 (10)
C13	0.0294 (10)	0.0205 (10)	0.0265 (10)	−0.0013 (8)	0.0053 (9)	0.0053 (8)
C14	0.0354 (12)	0.0251 (11)	0.0305 (11)	−0.0060 (9)	0.0127 (9)	0.0061 (9)
C15	0.0397 (12)	0.0252 (11)	0.0257 (11)	−0.0046 (9)	0.0157 (9)	−0.0004 (9)
O16	0.0432 (9)	0.0231 (8)	0.0287 (8)	−0.0081 (6)	0.0108 (7)	0.0021 (6)
C17	0.0365 (12)	0.0225 (11)	0.0343 (12)	−0.0059 (9)	0.0070 (10)	0.0075 (9)

### Geometric parameters (Å, °)

**Table:**

Cu1—N6^i^	1.9403 (16)	C9—C10	1.454 (3)
Cu1—N6	1.9403 (16)	C10—C15	1.386 (3)
Cu1—N1^i^	1.9451 (17)	C10—C11	1.398 (3)
Cu1—N1	1.9451 (17)	C11—C12	1.380 (3)
N1—C2	1.311 (3)	C11—H11	0.95
N1—H1	0.88	C12—C13	1.394 (3)
C2—N4	1.346 (3)	C12—H12	0.95
C2—C3	1.515 (3)	C13—O16	1.361 (2)
C3—H3A	0.98	C13—C14	1.390 (3)
C3—H3B	0.98	C14—C15	1.388 (3)
C3—H3C	0.98	C14—H14	0.95
N4—C5	1.329 (3)	C15—H15	0.95
C5—N6	1.324 (3)	O16—C17	1.436 (3)
C5—O7	1.370 (2)	C17—H17A	0.98
N6—N8	1.405 (2)	C17—H17B	0.98
O7—C9	1.377 (2)	C17—H17C	0.98
N8—C9	1.289 (2)		
			
N6^i^—Cu1—N6	180.00 (6)	N8—C9—C10	127.92 (19)
N6^i^—Cu1—N1^i^	87.39 (7)	O7—C9—C10	119.18 (17)
N6—Cu1—N1^i^	92.61 (7)	C15—C10—C11	118.54 (19)
N6^i^—Cu1—N1	92.61 (7)	C15—C10—C9	121.09 (19)
N6—Cu1—N1	87.39 (7)	C11—C10—C9	120.36 (19)
N1^i^—Cu1—N1	180	C12—C11—C10	120.5 (2)
C2—N1—Cu1	131.13 (14)	C12—C11—H11	119.7
C2—N1—H1	114.4	C10—C11—H11	119.7
Cu1—N1—H1	114.4	C11—C12—C13	120.2 (2)
N1—C2—N4	125.48 (18)	C11—C12—H12	119.9
N1—C2—C3	120.38 (18)	C13—C12—H12	119.9
N4—C2—C3	114.13 (17)	O16—C13—C14	124.78 (19)
C2—C3—H3A	109.5	O16—C13—C12	115.32 (19)
C2—C3—H3B	109.5	C14—C13—C12	119.90 (19)
H3A—C3—H3B	109.5	C15—C14—C13	119.2 (2)
C2—C3—H3C	109.5	C15—C14—H14	120.4
H3A—C3—H3C	109.5	C13—C14—H14	120.4
H3B—C3—H3C	109.5	C10—C15—C14	121.6 (2)
C5—N4—C2	118.09 (17)	C10—C15—H15	119.2
N6—C5—N4	133.31 (18)	C14—C15—H15	119.2
N6—C5—O7	109.26 (17)	C13—O16—C17	117.41 (17)
N4—C5—O7	117.43 (17)	O16—C17—H17A	109.5
C5—N6—N8	108.50 (16)	O16—C17—H17B	109.5
C5—N6—Cu1	124.23 (14)	H17A—C17—H17B	109.5
N8—N6—Cu1	126.65 (13)	O16—C17—H17C	109.5
C5—O7—C9	104.03 (14)	H17A—C17—H17C	109.5
C9—N8—N6	105.28 (16)	H17B—C17—H17C	109.5
N8—C9—O7	112.90 (17)		
			
N6^i^—Cu1—N1—C2	−177.96 (19)	N6—N8—C9—O7	0.9 (2)
N6—Cu1—N1—C2	2.04 (19)	N6—N8—C9—C10	−178.20 (19)
Cu1—N1—C2—N4	1.8 (3)	C5—O7—C9—N8	−1.4 (2)
Cu1—N1—C2—C3	−178.05 (14)	C5—O7—C9—C10	177.76 (17)
N1—C2—N4—C5	−2.6 (3)	N8—C9—C10—C15	−170.3 (2)
C3—C2—N4—C5	177.23 (17)	O7—C9—C10—C15	10.7 (3)
C2—N4—C5—N6	−2.5 (3)	N8—C9—C10—C11	9.8 (3)
C2—N4—C5—O7	177.53 (17)	O7—C9—C10—C11	−169.16 (19)
N4—C5—N6—N8	179.1 (2)	C15—C10—C11—C12	0.2 (4)
O7—C5—N6—N8	−0.9 (2)	C9—C10—C11—C12	180.0 (2)
N4—C5—N6—Cu1	7.6 (3)	C10—C11—C12—C13	0.7 (4)
O7—C5—N6—Cu1	−172.39 (12)	C11—C12—C13—O16	179.4 (2)
N1^i^—Cu1—N6—C5	174.14 (16)	C11—C12—C13—C14	−1.1 (4)
N1—Cu1—N6—C5	−5.86 (16)	O16—C13—C14—C15	−179.9 (2)
N1^i^—Cu1—N6—N8	4.22 (16)	C12—C13—C14—C15	0.6 (3)
N1—Cu1—N6—N8	−175.78 (16)	C11—C10—C15—C14	−0.6 (3)
N6—C5—O7—C9	1.4 (2)	C9—C10—C15—C14	179.6 (2)
N4—C5—O7—C9	−178.66 (17)	C13—C14—C15—C10	0.2 (4)
C5—N6—N8—C9	0.0 (2)	C14—C13—O16—C17	0.6 (3)
Cu1—N6—N8—C9	171.26 (14)	C12—C13—O16—C17	−179.9 (2)

Symmetry codes: (i) −*x*, −*y*, −*z*+1.

### Hydrogen-bond geometry (Å, °)

**Table:**

*D*—H···*A*	*D*—H	H···*A*	*D*···*A*	*D*—H···*A*
N1—H1···N8^i^	0.88	2.42	2.983 (2)	123

Symmetry codes: (i) −*x*, −*y*, −*z*+1.
